# The effect of substrate temperature and oxygen partial pressure on the properties of nanocrystalline copper oxide thin films grown by pulsed laser deposition

**DOI:** 10.1016/j.dib.2020.106644

**Published:** 2020-12-13

**Authors:** Syed Farid Uddin Farhad

**Affiliations:** a H.H. Wills Physics Laboratory, School of Physics, University of Bristol, BS8 1TL, UK; b Industrial Physics Division, BCSIR Labs, Dhaka, Bangladesh Council of Scientific and Industrial Research (BCSIR), Dhaka 1205, Bangladesh

**Keywords:** Pulsed Laser Deposition (PLD), Copper oxide thin film, Bi-axial stress, Dynamic photo-transient current, ZnO/Cu_2_O based junction, Transmission Electron Microscopy (TEM), Energy dispersive x-ray (EDX)

## Abstract

The data presented in this paper are related to the research article entitled “Pulsed laser deposition of single phase n- and p-type Cu_2_O thin films with low resistivity” (S.F.U. Farhad et al., 2020) [1]. The detailed processing conditions of copper oxide thin films and a variety of characterization techniques used are described in the same ref. [1]https://doi.org/10.1016/j.matdes.2020.108848. Thin films need to grow on different substrates to elucidate various properties of the individual layer for attaining optimum processing conditions required for devising efficient optoelectronic junctions as well as thin film stacks for different sensing applications. This article describes the effect of substrate temperature and oxygen partial pressure on the structural, morphological, optical, and electrical properties of pulsed laser deposited (PLD) nanocrystalline copper oxide thin films on quartz glass, ITO, NaCl(100), Si(100), ZnO coated FTO substrates. The low temperature grown copper oxide and zinc oxide thin films by PLD were used for devising solid n-ZnO/p-Cu_2_O junction and investigated their photovoltaic and interface properties using dynamic photo-transient current measurement at zero bias voltage and TEM/EDX respectively. These datasets are made publicly available for enabling extended analyses and as a guide for further research.

## Specifications Table

**Table:** 

Subject	Materials Engineering, Physics, Electrochemistry
Specific subject area	Metal oxide semiconductors for optoelectronic applications
Type of data	Tables; Images; Figures
How data were acquired	X-ray Diffraction (XRD) (Bruker AXS D8 Advance), FE-SEM (JEOL JSM 6330F), Focused Ion Beam (FIB), TEM (JEOL 2010), Renishaw 2000 confocal Raman and Photoluminescence spectrometer (λ_ext_ = 514.5 nm Ar-ion laser (P ≤ 5 mW)), UV-VIS-NIR spectrophotometer coupled with an integrating sphere (Shimadzu UV2600 ISP), Keithley 2400 and 2450 source-measure-unit (SMU), Keithley 6221 AC/DC precision current source coupled with a Nanovoltmeter (Keithley 2182A), 1 Tesla permanent magnet (Magnetsales UK Ltd.), Variable Angle Spectroscopic Ellipsometry (VASE) (M-2000 U, J.A. Woollam Co.), Potentiostat/Galvanostat (IVIUM CompactStat) equipped with a frequency analyzer (FRA).
Data format	Raw and analyzed
Parameters for data collection	Deposited copper oxide thin films on quartz glass, ITO, NaCl (100), ZnO (PLD)/FTO etc. were characterized without further processing, except one set of films that annealed in air at 550 °C for 1 h for comparison purposes. VASE: Reflection mode, Optical diffuse reflectance: Copper oxides on quartz glass.
Description of data collection	Deposition of copper oxide and zinc oxide thin films on variety of substrates by Pulsed Laser Deposition (PLD). Characerization of as-grown and annealed thin films by variety of techniques and photovoltaic response measurement of solid p-Cu_2_O/n-ZnO junctions. The raw data for the figures can be found in the Mendeley dataset.
Data source location	University of Bristol, Bristol BS8 1TS, United Kingdom Industrial Physics Division (IPD), BCSIR Labs, Dhaka 1205, Bangladesh
Data accessibility	https://data.mendeley.com/datasets/rmzrnz2nd7/3
Related research article	S.F.U. Farhad, D. Cherns, J. A. Smith, N. A. Fox, and D. J. Fermín, Pulsed laser deposition of single-phase n- and p-type copper oxide thin films with low resistivity, Materials & Design, 193 (2020), 108848. https://doi.org/10.1016/j.matdes.2020.108848

## Value of the Data

• Nanocrystalline copper oxide thin films were grown by PLD on amorphous, polycrystalline, and crystalline substrates at relatively low temperatures (≤ 300 °C) and wide range of oxygen partial pressures to attain thin films with tuneable structural, optical, and electrical properties.
• Good quality copper oxide thin films attainable at low processing temperatures are desirable for realizing optoelectronic devices requiring low thermal budget. Researchers who are interested in Cu_2_O based thin film solar cell, p-channel thin film transistor (TFT), and different sensing devices can also be benefited from these data. In literature, most of Cu_2_O thin films grown by physical vapor deposition with processing temperature 500 °C and above to attain desired structural, optical and electrical properties. Such high processing temperature may not be suitable for practical applications and would be difficult to integrate the film stacks on conventional soda lime glass (SLG) and other technologically important substrates. Therefore, the methodology used to deposit and characterize the pristine and processed samples as well as the base values of physical properties attained through the optimization of wide parameter space can be used for comparison to data reported by others.
• The room-temperature grown Cu_2_O thin films showed rectification and a photovoltaic (PV) response while making a junction with ZnO previously grown by PLD at 300 °C.
• The cross-sectional thin foils of the ZnO/Cu_2_O interface was made by a focused ion beam (FIB) assisted FE-SEM using the in situ liftout technique and investigated by TEM and EDX.

## 1 Data Description

**Fig. 1:**
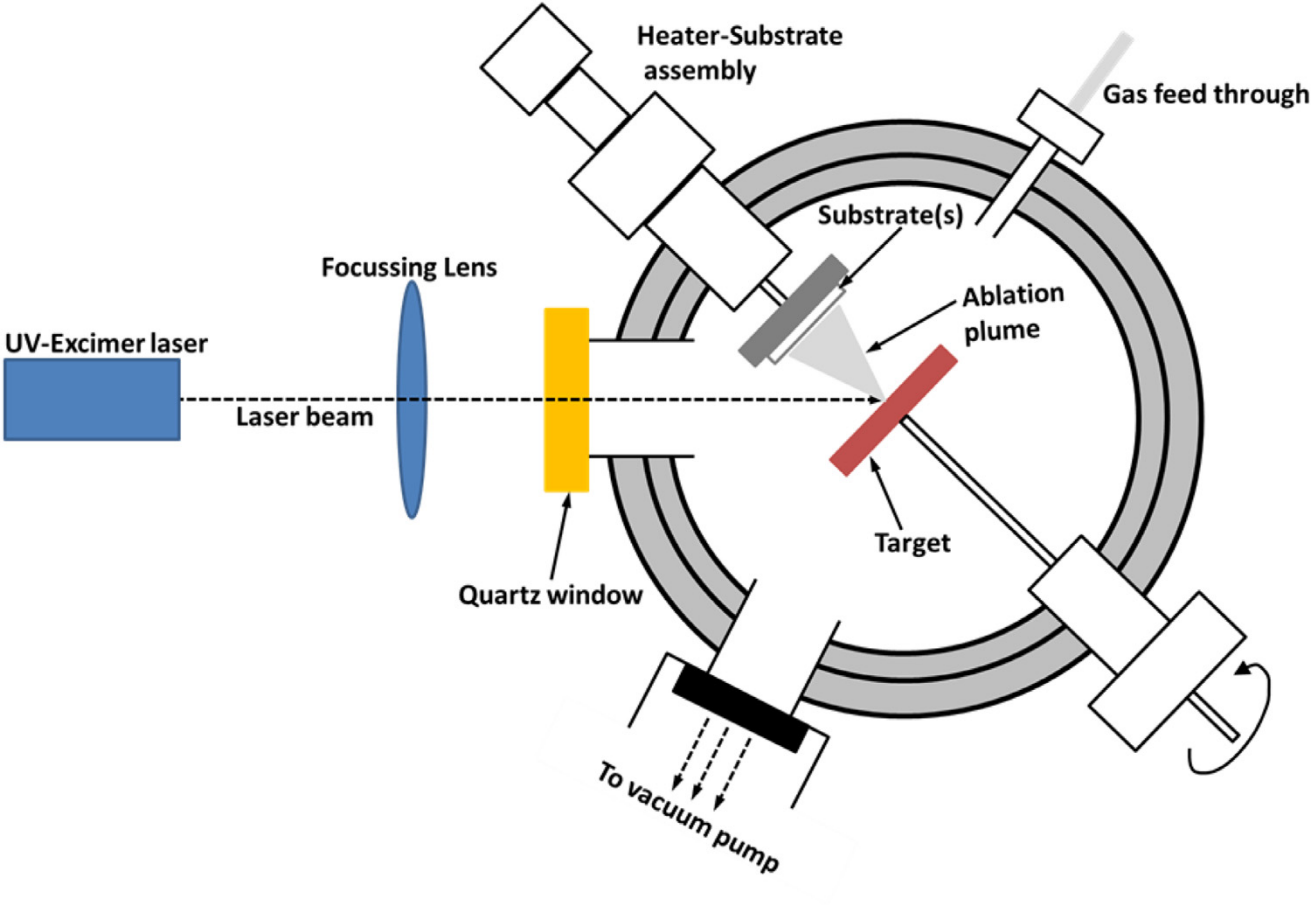
Schematic diagram of the PLD setup used for depositing copper oxide and zinc oxide thin films in this study.

Table 1 summarizes the important PLD processing conditions (substrate temperature (T_sub_), oxygen partial pressure (O_2pp_), and Laser energy per pulse (LP)) for the growth of single phase copper (I) oxide (Cu_2_O) thin film with controlling exposed crystal surfaces as well as type and level of conductivity. Fig. 1 shows the PLD setup with the arrangement of substrate holder to deposit copper oxide (and also zinc oxide) thin films simultaneously on two different substrates (see Fig. S7a and Fig. S7b of ref. [1]). The XRD *Ɵ* – 2 *Ɵ* (out-of-plane) plot of copper oxide films deposited on quartz substrates at 25 °C ≤ T_sub_ ≤ 400 °C with constant O_2pp_ ≈ 10 mTorr and O_2pp_ ≈ 3 mTorr can be found in Fig. S1a and Fig. S1b respectively in the supplementary material of ref. [1]. The estimated phase fraction [3] of copper oxide films based on XRD data are summarized in Table 2.

**Table 1:** PLD process conditions used for producing copper oxide thin film. Base vacuum of PLD chamber ≤ 10^−6^ mTorr; Target substrate distance ∼ 5 cm

Process parameter → Investigating effect ↓	T_sub_ (°C)	O_2pp_ (mTorr)	LP (mJ) (spot size ∼ 0.18 × 0.09 cm^2^)
Growth temperature	25 – 400	10	25
	25 – 300	3	
Background gas pressure	25	0 – 7	25
	200	0 – 7	
Substrates	With constant T_sub_, O_2pp_ and LP		
	Crystalline: NaCl(100), and Si(100)	Polycrystalline (conducting):ITO, FTO, ZnO, AZO	Amorphous (insulating): Quartz glass

**Table 2:** Copper oxide phases in the deposited films estimated from the XRD results shown in the Fig. S1 of ref. [1]. Optimum deposition conditions for phase pure Cu_2_O are highlighted (Bold font).

	Copper oxide phase fraction (%)						
	O_2pp_ ≈ 10 mTorr			O_2pp_ ≈ 3 mTorr			
T_sub_ (°C)	Cu	Cu_2_O	CuO	Cu	Cu_2_O	CuO	Cu_x_O_y_
**25**	-	40	60	-	**100**	-	-
100	-	48	52	1*	55	-	44
**200**	-	93	7	-	**85**	-	15
300	11	89	-	1	34	-	65
400	6	94	-				
Ann@550 ^0^C	-	-	100	-	-	100	-

⁎ Deconvolution resulted a peak at *2θ* ≈ 43.42^0^ which could be assigned to Cu (111)

**Fig. 2:**
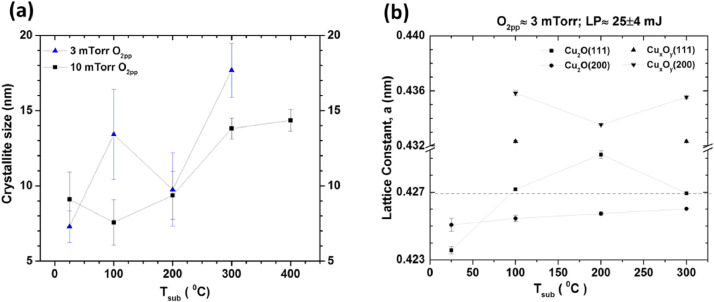
The average crystalline domain size (a) and lattice constant (b) variation as a function of substrate temperature. The horizontal dashed line in panel (b) indicates the bulk lattice constant value (*a* ≈ 0.427 nm) of pure copper oxide.

Thin films deposited using oxygen rich conditions (i.e., O_2pp_ ≈ 10 mTorr) show a mixture of CuO and Cu_2_O phase but no evidence of Cu_4_O_3_ phase is obtained. In contrast, thin films deposited using oxygen poor conditions (i.e., O_2pp_ ≈ 3 mTorr) at T_sub_ ≈25 °C, exhibit strong Cu_2_O only with (111) and (200) but those grown at 100 °C ≤ T_sub_ ≤ 300 °C contains Cu_2_O phase along with Cu_x_O_y_ [2]. No standard samples are used for quantification, rather the relative amounts of phase fraction were estimated using ‘Inorganic Crystal Structures Database (ICSD)’ patterns as the basis for phase identification. The variation of average crystallite domain size and lattice constant of nanocrystalline copper oxides thin films as a function of substrate temperature are shown in Fig. 2a and 2b. For Cu_x_O_y_ phase, a defect structure of Cu_2_O [2], lattice parameters calculated using both (111) (denoted by ▲) and (200) (denoted by ▼) orientation found to be higher than the bulk (see Fig. 2b) and the estimated various strains were found to be ∼1% or more for films deposited at 100 °C ≤ T_sub_ ≤ 300 °C. The Bi-axial strain-stress of as-grown PLD films on quartz substrate were calculated from ex-situ XRD data analyses using the following relations [3], [4], [5]:

Elongation strain ($\varepsilon_{⊥}$) perpendicular to the plane of thin film,

(1) $$\varepsilon_{⊥}=\frac{\left(a-a_{0}\right)}{a_{0}}$$

(2) $$\text{For}\;\text{compressive}\;\text{stress},\;\varepsilon ∥\;=\;-\left(C11/2C12\right)\;\times \;\varepsilon_{⊥}$$

(3) $$\text{For}\;\text{tensile}\;\text{stress},\;\varepsilon ∥\;=\;-\;\left[C12/(C11+C12\right]\times \;\varepsilon_{⊥}$$

where, aand $a_{0}$ (∼0.427 nm [3]) are the lattice constants for thin film and bulk crystals respectively, C_ij_ are the elastic stiffness of the material of interest, for Cu_2_O: C_11_ ∼ 116.5 GPa and C_12_∼105.3 GPa; the corresponding stress, *σ*, is related by Hooke's law [5]:

(4) $$\sigma_{∥}^{Cu2O}=K_{Cu2O\;}\varepsilon_{∥}^{Cu2O}$$

Where, $K_{Cu2O}=\frac{\left(C_{11}-C_{12}\right)\left(C_{11}+2C_{12}\right)}{C_{11}}$ ≈ 31.5 GPa. The in-plane strain (as well as stress) induced in the PLD grown copper oxide films at various growth temperatures were calculated using equations (1) **–** (4) and summarized in Table 3. It is to be noted that XRD data were recorded at room temperature using Cu K_α_ (λ ≈ 1.5406 Ǻ) radiation. A step size of ∼0.025^0^ with 18 s per step was used and during scanning, samples were rotated to homogenize the measurements [1].

**Table 3:** Bi-axial strain-stress related calculations for cubic crystals of (111) and (200) reflection estimated from the XRD results shown in the Fig. S1b in the supplementary material of ref. [1]. Large compressive strains for Cu_x_O_y_ phase parallel to the substrate are highlighted (Bold font).

T_sub_ (°C)	Phase	*a*(111) (Å)	*a*(200) (Å)	${ɛ}_{⊥}$_(111)_ (±0.05) _(%)_	${ɛ}_{⊥}$_(200)_ (±0.05) _(%)_	ε_|| (111)_ (±0.05) _(%)_	ε_|| (200)_ (±0.05) _(%)_	σ_|| (111)_ (±0.05) _(GPa)_	σ_|| (200)_ (±0.05) _(GPa)_
25	Cu_2_O	4.24	4.25	-0.7	-0.5	0.4	0.3	12.6	9.5
100	Cu_x_O_y_	4.32	4.36	1.2	2.1	**-0.7**	**-1.3**	-18.9	-37.8
	Cu_2_O	4.27	4.25	0	-0.5	0	0.3	0	9.5
200	Cu_x_O_y_	-	4.34	-	1.6	-	**-1.0**	-	-28.4
	Cu_2_O	4.29	4.26	0.5	-0.2	-0.3	0.1	-9.5	3.2
300	Cu_x_O_y_	4.32	4.36	1.2	2.1	**-0.7**	**-1.3**	-18.9	-37.8
	Cu_2_O	4.27	4.26	0	-0.2	0	0.1	0	3.2

**Fig. 3:**
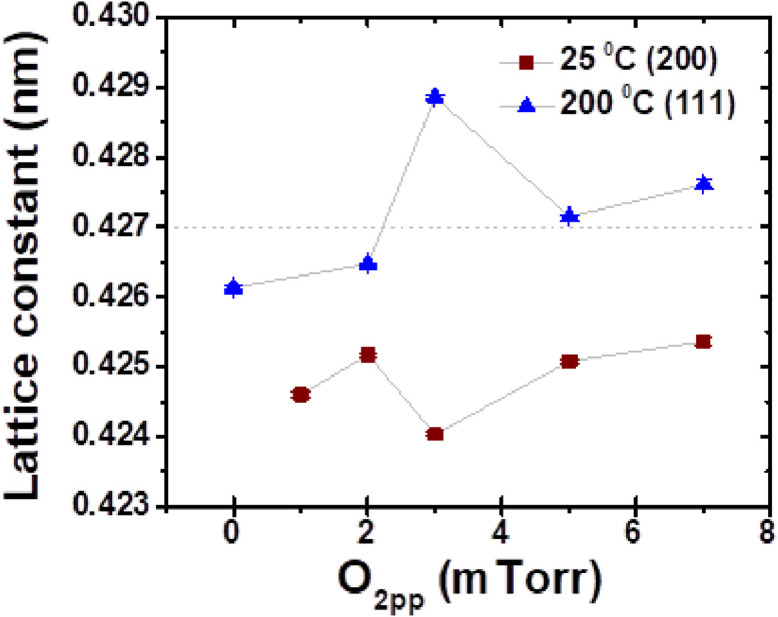
The variation of lattice constant of cuprous oxide films deposited at T_sub_ ≈ 25 °C (denoted by ■) and T_sub_ ≈ 200 °C (denoted by ▲) as a function of O_2pp_. The horizontal dashed line indicates the bulk lattice constant value (*a* ≈ 0.427 nm) of pure copper oxide.

For samples grown at T_sub_ ≈ 25 °C and T_sub_ ≈ 200 °C using O_2pp_ ≈ 0 –7 mTorr, the variation of lattice constant (*a*) (Fig. 3), average crystallite domain size (Fig. S3a) and texture coefficient (Fig. S3b) were analyzed [1]. The texturing coefficient (f) is calculated using Eq. (1) considering (111) and (200) are the only planes [3]:

(5) f=1−2y/(x+y)

where, $x=\frac{I(111)}{I(200)}$, $y=\frac{I_{0}\left(111\right)}{I_{0}\left(200\right)}$; I(hkl) and I_0_(hkl) are the intensities of (hkl) X-ray reflection planes of as grown thin films and bulk Cu_2_O respectively. (XRD of bulk Cu_2_O powder scrapped off from PLD target can be found in Fig. S1.1 of the supplementary material of ref. [1]). When f→-1, thin films are highly (200) textured and crystallites are dominantly (111) orientated when f→1.

**Fig. 4:**
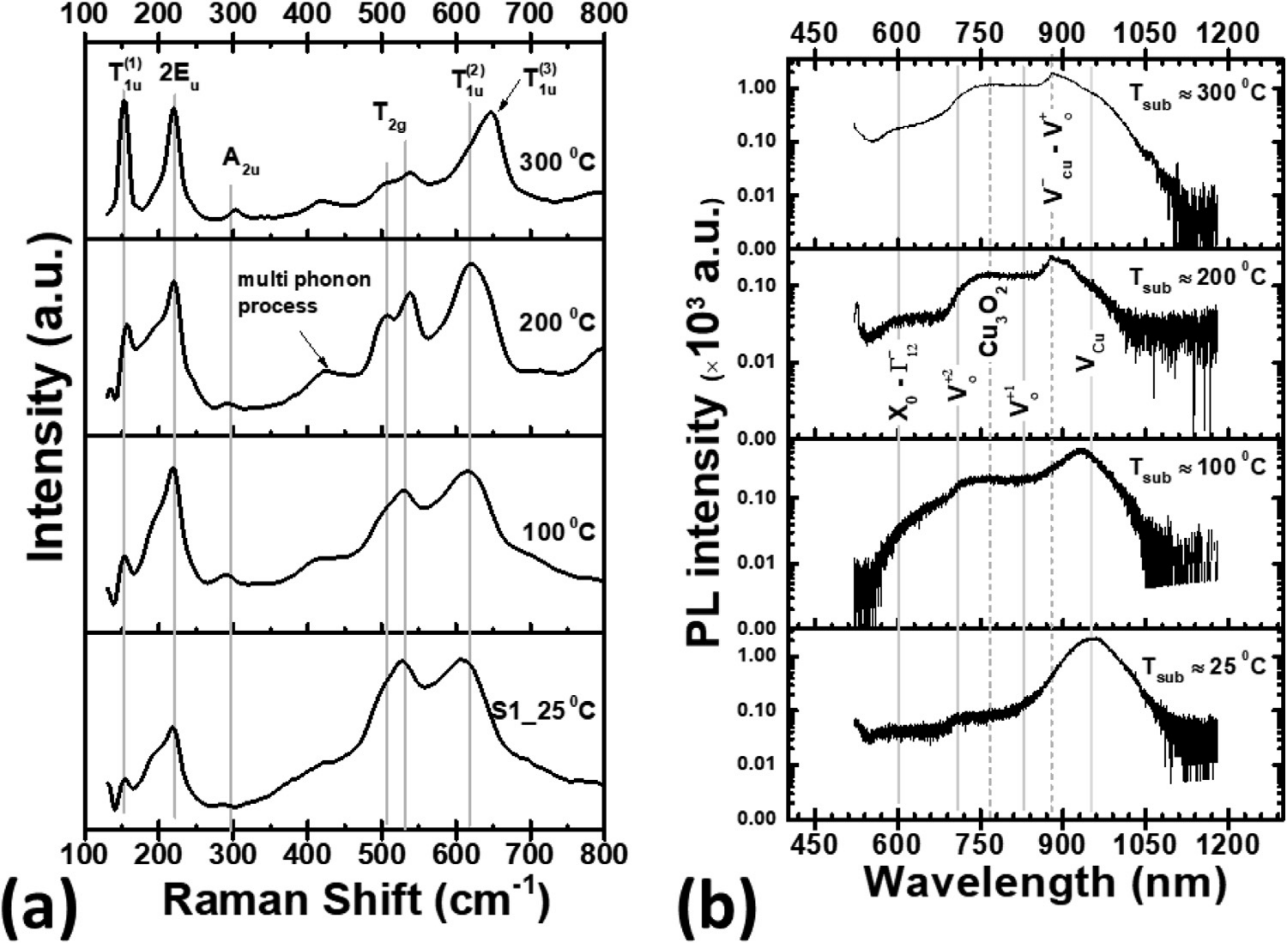
Room temperature Raman spectra (a) (reproduced from the supplementary material of ref. [1]) and Photoluminescence (PL) spectra (b) of copper oxide thin films deposited at T_sub_ ≈ 25 °C – 300 °C onto quartz substrate with a constant laser pulse energy (LP ≈ 25±4 mJ) and O_2pp_ ≈ 3 mTorr. The vertical lines indicate the reference vibrational modes and luminescence peaks of copper oxide [1], [2], [3].

The electrical and optical properties of the deposited thin films on quartz substrate can be found in ref. [1]. The room temperature Photoluminescence and Raman spectra of copper oxide thin films grown at T_sub_ ≈ 25 °C – 300 °C onto quartz substrate with a constant laser pulse energy (LP ≈ 25±4 mJ) and O_2pp_ ≈ 3 mTorr are shown in Fig. 4a and 4b respectively.

**Fig. 5:**
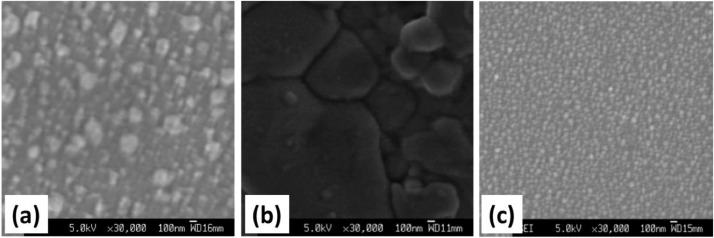
SEM micrographs of copper oxide thin films deposited on (a) ITO, (b) NaCl(100), and (c) quartz glass substrate using O_2pp_ ≈ 5 mTorr.

**Fig. 6:**
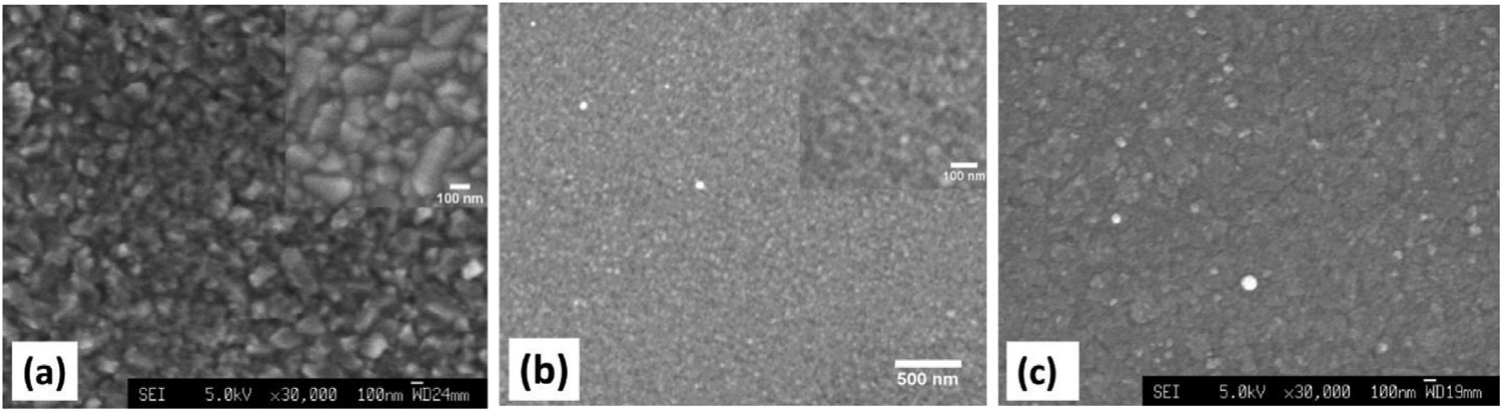
SEM micrographs of ZnO thin film grown on FTO substrate (a). The inset in (a) shows the surface morphology of the blank FTO. Al-doped ZnO (AZO) thin film on soda lime glass (b). The inset in (b) shows the zoomed area of the same sample. AZO thin film grown on ITO coated glass (c). Both ZnO and AZO thin films were also grown by PLD at O_2pp_ ≈ 10 mTorr _T_sub_ ≈ 300 °C and O_2pp_ ≈ 10 mTorr _T_sub_ ≈ 400 °C respectively.

The XRD patterns of room temperature grown Cu_2_O films on amorphous, polycrystalline and crystalline substrates can be found in the supplementary material of ref. [1]. The FE-SEM investigated surface morphologies of Copper-Oxide and Zinc Oxide thin films grown on different substrates at various deposition and processing conditions are presented respectively in Fig. 5, Fig. 6 below.

### 1.1 Low temperature PLD grown ZnO/Cu_2_O based solar cell

**Fig. 7:**
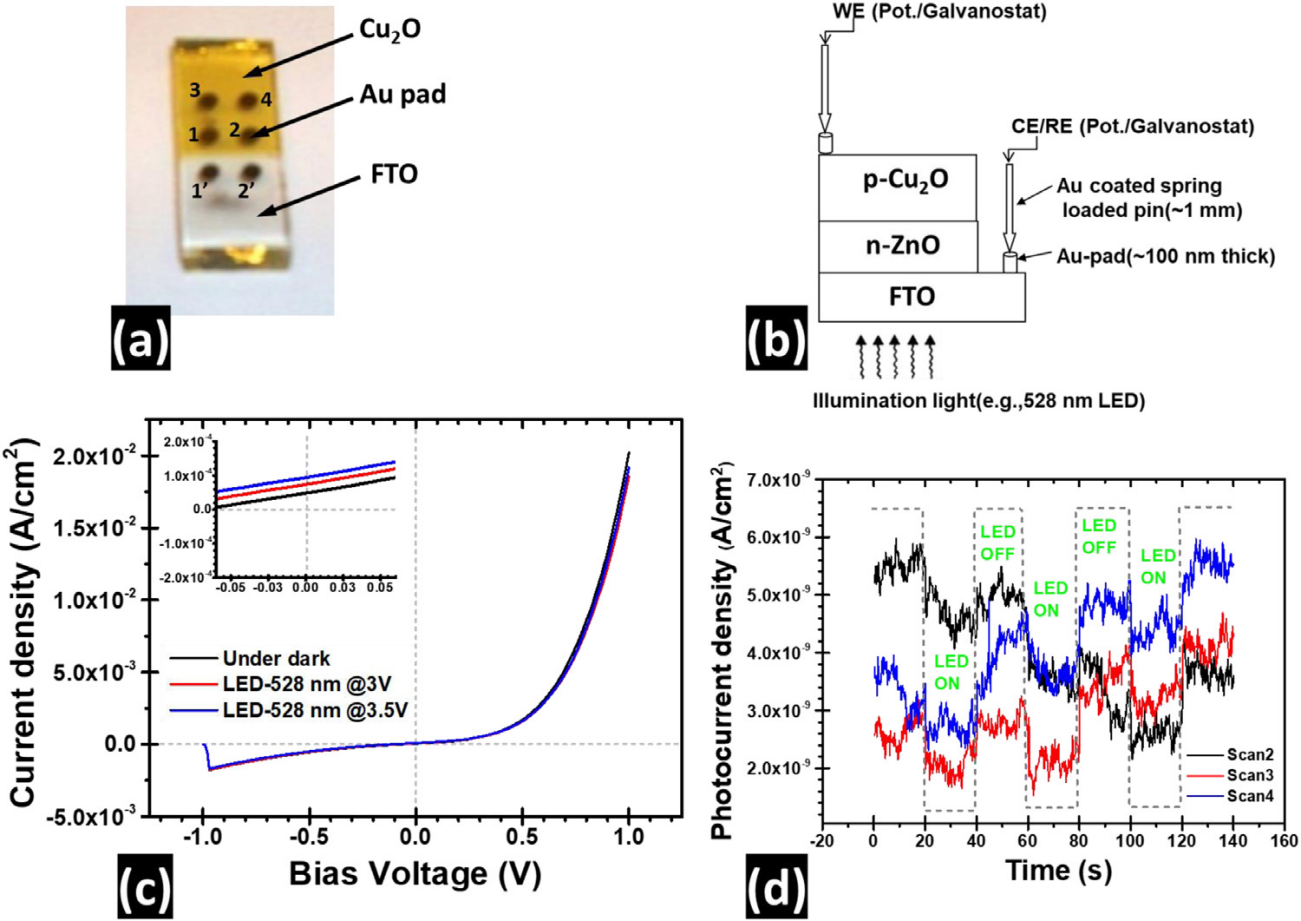
Photograph of a typical PLD-grown solar cell of Au/FTO/ZnO/Cu_2_O/Au structure (a); A schematic of the photocurrent measurement setup using the IVIUM potentiostat/galvanostat as a source measure unit (SMU) (b). The characteristic J-V curve of under dark and under 528 nm-LED illumination (c), a zoomed area of the J-V curve near zero bias voltage shown in the inset of (c). Transient photocurrent of the same cell measured under periodic LED illumination (d), where ‘On’ and ‘Off’ steps of the transient photocurrent is demonstrated by the dotted curve to assist the reader.

A set of four thin film solid heterojunctions were fabricated on commercially available FTO coated glass substrates by successive deposition of ZnO layer (T_sub_ ≈ 300 °C) followed by a Cu_2_O thin layer (T_sub_ ≈ 25 °C). Prior to the deposition of Cu_2_O, PLD grown ZnO layer was subject to anneal at 366 °C inside the PLD chamber (Vacuum < 10^−6^ mBar) for 40 min. (The vacuum of PLD chamber was interrupted in order to change ablation target). Six circular gold (Au) pads (∼100 nm thick and 2 mm dia, 2 mm distance apart, see the picture in the Fig. 7a) were deposited by a thermal evaporator under high vacuum (<10^−6^ mTorr) through a patterned shadow mask to make good ohmic contacts. Therefore, the final device has an architecture that comprised the Au/FTO/ZnO/Cu_2_O/Au thin film stacks. The electrical connection of the p- and n-type metal oxide sides were made to the source meter (e.g., Potentiostat) probes via Au coated spring-loaded pins (tip dia∼1 mm) to avoid scratching film surface and/or short circuiting between the metal oxide layers. A typical device structure based on Cu_2_O/ZnO heterojunction and its electrical contact with the potentiostat is shown in Fig. 7b. Current-Voltage and Transient current measurements were performed in the Dark, and under 528 nm LED illumination trough the FTO side (see Fig. 7b and 7c). An IVIUM-CompactStat potentiostat was used as a source measure unit (SMU). The working electrode (WE) and short-circuited counter electrode/reference electrode (CE/RE) of the potentiostat were treated as positive and negative end of a voltage source meter respectively [3].

Fig. 7a shows a photograph of a typical PLD grown solar cell with Au-FTO/ZnO/Cu_2_O/Au thin film stacks. The Au pads on FTO and Cu_2_O layers are numbered to assist the reader. Two adjucent gold (Au∼2 mm dia, 100 nm thick) pads in the same layer were used for measuring contact behaviour of FTO (1′ and 2′ on white part in 7a) and p-Cu_2_O (yellow part in 7a) layer with Au (see supplimentary materials of ref. [1] for details).

A schematic of the J-V curve measurement setup is shown in Fig. 7b. In Fig. 7c, the dark and LED (528 nm) modulated J-V characteristics curve of Au-FTO/n-ZnO/p-Cu_2_O/Au cell shows stable rectifying behavior of Cu_2_O-ZnO system (Au-FTO-1′ & Au-Cu_2_O-3 contact). Higher illumination of the LED (by sourcing 3 V and 3.5 V voltage to the 528 nm-LED) on the cell produced higher photo responses (see Fig. 7c inset). The dynamic photo response at zero bias voltage of another junction area of the Au-FTO/n-ZnO/p-Cu_2_O/Au cell (Au-FTO- 2′ & Au-Cu_2_O-4 contact) switching with the LED (wavelength just above the band gap of the absorber Cu_2_O (E_g_ ≈ 2.2 eV (564 nm)) layer) by a pulse width of 20 s exhibited very low and noisy photocurrent yet distinguishable from the dark current (see Fig. 7d). The photo responses of the cell were seen to degrade over time (see scan 2, scan 3, and scan 4 in Fig. 7d).

### 1.2 Morphology, structure, and chemical composition of the ZnO/ Cu_2_O interface

**Fig. 8:**
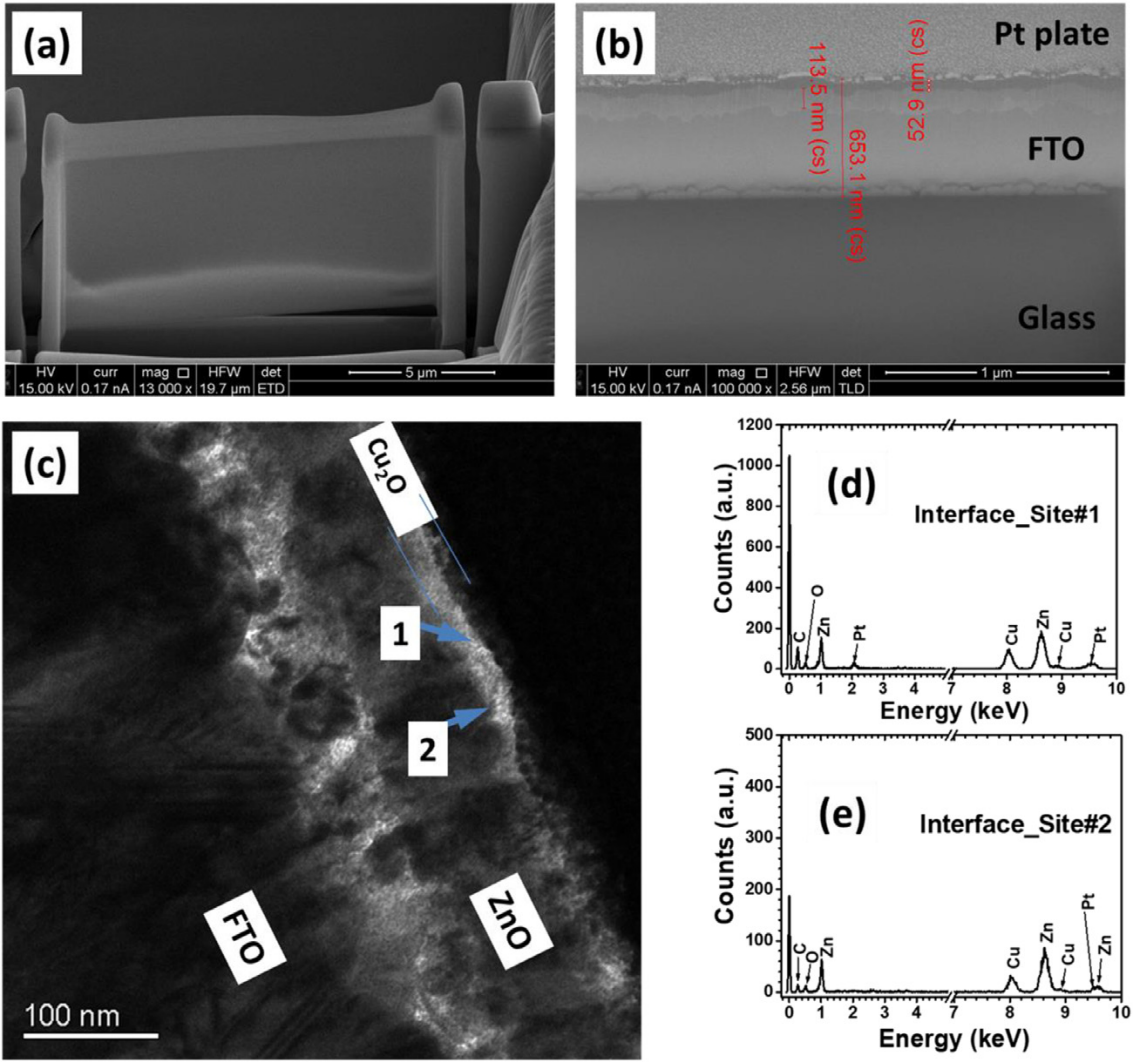
Focused Ion Beam (FIB) assisted cross-sectional specimen preparation of Cu_2_O/ZnO stack (a); SEM micrograph of the same FIB specimen (b): Cu_2_O (∼53 nm), and ZnO (∼114 nm) layer. Platinum (Pt)-plate was used to protect thin layers during FIB cross-sectional specimen preparation. TEM Bright field image of the FIB cross-sectional sample (c): The EDX spectra of the amorphous interface layer between ZnO and Cu_2_O at site#1 (d) and site#2 (e) (marked by arrows in c).

The interface quality between ZnO and Cu_2_O layer of the PLD grown n-ZnO/p-Cu_2_O stack was also investigated by SEM and TEM. The SEM micrograph of the FIB assisted cross-sectional Cu_2_O/ZnO specimen (see Fig. 8a) revealed a very thin but continuous Cu_2_O (∼53 nm) and ZnO (∼114 nm) layer across the specimen (see Fig. 8b). The TEM bright field (BF) image of the FIB specimen revealed an amorphous layer (marked by arrows, SAED pattern not shown here) between the Cu_2_O and ZnO layer (see Fig. 8c) (see also Fig. S13 in the supplementary materials of ref. [1]). A subsequent energy dispersive x-ray (EDX) microanalysis of the Cu_2_O/ZnO interface at two different sites (site#1 and site#2 are marked by arrows in Fig. 8c) revealed the presence of O, Zn, and Cu elements (see Fig. 8d and 8e).

## 2 Experimental Design, Materials and Methods

All experimental design, materials, and methods were based on reported paper [1].

### 2.1 Target materials

The target material in PLD was commercially available sintered ceramic Cu_2_O (purity∼99.95%). Although, single crystals are more preferable in the case of choosing target material, however, for many materials, they are difficult to obtain. The majority of the reported literature on copper oxide thin film deposition used CuO ceramic target, although Cu_2_O as a target material also reported by few researchers. We prefered Cu_2_O over CuO as a target material for the following reasons: Cu_2_O has higher absorption coefficient, lower thermal expansion coefficient, lower boiling point, and lower melting point than that of CuO (see Table 4 below). Target-material having high thermal expansion coefficient and high melting point has been reported to have high probability of ‘exfoliation’ during laser ablation due to the fact that “the thermal oscillations induced by repeated laser excitation do not exceed melting point” of the target-material ([3] and refs. therein). For ZnO and Al-doped ZnO (AZO) thin films, ceramic tragets with purity∼99.999% and ∼99.999% (composed of 99 wt% ZnO and 1 wt% Al_2_O_3_) were used respectively.

**Table 4:** Crystallographic and physical properties of Cu_2_O and CuO ([3,[6], [7], [8], [9], [10], [11], [12]]).

Properties	Cu_2_O (cubic) (space group: $\mathrm{Pn}\bar{3}m,\;O_{h}^{4}$)	CuO (monoclinic) (space group: **C2/c(#15)**)
Lattice constant	4.2696±0.0010 Å	*a =* 4.6837 Å *b =* 3.4226 Å *c =* 5.1288 Å *β = 99.54^0^* α = γ = 90^0^
Cu–O bond length	1.85 Å	1.96 Å
O–O separation	3.68 Å	2.62 Å
Cu–Cu separation	3.02 Å	2.90 Å
Density	6.10 g/cm^3^	6.52 g/cm^3^
Molar mass, M	143.14 g/mol	79.57 g/mol
Melting point	1235 ^0^C	1326 ^0^C[12]
Boiling point	1800 ^0^C	2000 ^0^C[12]
Young's modulus	30.12 GPa	81.6 GPa
Thermal expansion coefficient	2.3 × 10^−7^ K^−1^ (283 K)	(2– 6) × 10^−6^ K^−1^ (200 K)[8]
Dielectric constant	ε(0) = 7.11 and ε(∞) = 6.6	ε(0) ≈ 8.00 and ε(∞) = 6.45
Electron affinity (300 K)	∼ 3.1 eV(300 K)	∼ 4.07 eV
Work function	∼ 4.84 eV (300 K)	∼ 5.25 eV
Thermal conductivity	5.2 W/(Km)	8.6 W/(Km)
Specific heat capacity (300 K)	70 J/(K mol)	42.36 J/(K mol) [11]

### 2.2 Laser wavelength and processing parameters of the PLD setup

Bulk of the reported literature on copper oxide thin film deposition is focused on the use of KrF: *λ* = 248 nm excimer laser whereas use of the visible lasers (e.g., Nd: YAG laser operated at *λ* = 532 nm) is really scarce ([3] and refs. therein). This is due to the fact that thin film deposition using a longer wavelength (λ) laser is found to generate more droplets than a shorter wavelength laser. Furthermore, droplet density on the surface of the deposited films has been reported to be reduced as the optical absorption coefficient (α) increases. And as α increases with decreasing λ, therefore use of UV lasers (e.g., ArF:*λ* = 193 nm) generally encourages smoother film morphologies. Therefore, following laser and processing conditions are chosen for depositing copper oxide thin films: UV-ArF Excimer Laser (λ: 193 nm), repetition rate: 10 Hz, pulsed width: 20 ns; typical pulse energy: ∼25–30 mJ which, when focused onto the target, produced a laser fluence (LF) of ∼1.5 J/cm^2^– 2.0 J/cm^2^. The oxygen partial pressure (O_2pp)_ and substrate temperature (T_sub_) were varied to allow film growth in a stable regime, where no decomposition of the films is observed. The same PLD setup and operation conditions but with a fixed O_2pp_ = 10 mTorr; LF∼2.0 J/cm^2^ were used for depositing both ZnO and AZO thin films on various substrates.

## Credit Author Statement

**Syed Farid Uddin Farhad**: Conceptualization, Investigation, Data curation, Writing, editing and reviewing the manuscript.

## Declaration of Competing Interest

The author(s) declare that they have no known competing financial interests or personal relationships which have, or could be perceived to have, influenced the work reported in this article.
